# pH-Driven Reversible Assembly and Disassembly of Colloidal Gold Nanoparticles

**DOI:** 10.3389/fchem.2021.675491

**Published:** 2021-04-29

**Authors:** Yun Liu, Weihua Fu, Zhongsheng Xu, Liang Zhang, Tao Sun, Mengmeng Du, Xun Kang, Shilin Xiao, Chunyu Zhou, Mingfu Gong, Dong Zhang

**Affiliations:** ^1^Department of Radiology, Xinqiao Hospital, Army Medical University, Chongqing, China; ^2^Department of Urology, Xinqiao Hospital, Army Medical University, Chongqing, China

**Keywords:** gold nanoparticles, reversible self-assembly, pH-responsive, plasmonic, 3-aminopropyltriethoxysilane

## Abstract

Owing to the localized surface plasmon resonance (LSPR), dynamic manipulation of optical properties through the structure evolution of plasmonic nanoparticles has been intensively studied for practical applications. This paper describes a novel method for direct reversible self-assembly and dis-assembly of Au nanoparticles (AuNPs) in water driven by pH stimuli. Using 3-aminopropyltriethoxysilane (APTES) as the capping ligand and pH-responsive agent, the APTES hydrolyzes rapidly in response to acid and then condenses into silicon. On the contrary, the condensed silicon can be broken down into silicate by base, which subsequently deprotonates the APTES on AuNPs. By controlling condensation and decomposition of APTES, the plasmonic coupling among adjacent AuNPs could be reversible tuned to display the plasmonic color switching. This study provides a facile and distinctive strategy to regulate the reversible self-assembly of AuNPs, and it also offers a new avenue for other plasmonic nanoparticles to adjust plasmonic properties *via* reversible self-assembly.

## Introduction

Noble metal nanoparticles have been intensively studied for a wide range of applications because of the localized surface plasmon resonance (LSPR), which is strongly dependent on nanoparticle size, shape, and composition (Gao et al., 2014; Chen et al., 2016). Low-dimensional plasmonic nanoparticle assemblies with new optical properties have recently attracted considerable attention because of the near-field coupling between adjacent particles (Liu D. et al., 2018; Li and Yin, 2019; Li et al., 2020a). The ideal way is the reversible assembly of such plasmonic nanostructures, which could enable dynamic tuning of the surface plasmon coupling by responding the external stimuli, and therefore by taking advantage of the ultrasensitive gap-dependent properties of plasmonic coupling, they have great promises for applications such as colorimetric sensors, bio- and chemical detection, and therapeutics (Bonacchi et al., 2016; Pillai et al., 2016; Liu L. et al., 2018; Zhou et al., 2021). Recent studies have demonstrated the reversible assembly by controlling the nanoparticle separation *via* various methods, for example, by modulating solvent composition to change the ionic strength of the solution, adding moisture, thermo-, photo-, magnetical-, and pH-responsive ligands, or reversible linking molecules (such as DNA) (Liu et al., 2012; Liu L. et al., 2019; Ding et al., 2016; Fan et al., 2016; He et al., 2016, 2019; Grzelczak et al., 2019; Li et al., 2020b; Severoni et al., 2020). However, these methods mentioned above more or less remain shortages, for example, the solutions usually contain two or more solvents, the responsive ligands (thiol molecules, biological molecules, or polymers) usually need complicated organic synthesis, and the reversible assembly process is not sensitive and robust. It is therefore highly worthy to design simple and effective strategies toward reversible assembly and dynamic tuning of the plasmonic coupling properties of noble metal nanoparticles.

Herein, for the first time, we develop a novel method for direct reversible assembly of Au nanoparticles (AuNPs) in water driven by pH stimuli with robust dynamic tuning of the surface plasmonic coupling among AuNPs. By using 3-aminopropyltriethoxysilane (APTES) as the capping ligand and pH-responsive agent, the APTES hydrolyzes rapidly in response to acid and then condenses into silicon. On the contrary, the condensed silicon can be broken down into silicate by base, which subsequently deprotonate the APTES on AuNPs. By controlling condensation and decomposition of APTES, the plasmonic coupling among adjacent AuNPs could be reversible tuned to display the plasmonic color switching. This study provides a facile and distinctive strategy to regulate the reversible self-assembly of AuNPs, and it also offers a new avenue for other plasmonic nanoparticles to adjust plasmonic properties *via* reversible self-assembly.

## Materials and Methods

### Materials

Hydrogen tetrachloroaurate (III) trihydrate (HAuCl^−^_4_·3H_2_O) was purchased from Acros Organics, trisodium citrate (Na_3_C_6_H_5_O_7_·2H_2_O) and sodium hydroxide (NaOH) were obtained from Sino-pharm, ethanol (99.7%) from Adamas-beta. 3-Aminopropyltriethoxysilane (APTES) used here was purchased from Fisher Scientific. All chemicals were analytic grade and used without further purification. All solutions were prepared in deionized water (DI water, 18.2 MΩ·cm) from a Thermo Scientific Nanopure water purification system.

### Synthesis of Gold Nanoparticles

Colloidal AuNPs with an average diameter of 15 nm were synthesized by the classical sodium citrate-reduction method proposed by Turkevich et al. (1951). Typically, 20 μl of 2.5 mol/L chloroauric acid solution was added to boiling DI water (95 ml) with stirring, followed by the addition of an aqueous solution of trisodium citrate (5 ml, 1 wt%). The reaction was refluxed for 15 min, and the solution gradually turned from colorless to wine red. The AuNPs were isolated by centrifugation, washed with DI water two times, and dispersed in 10 ml of DI water. The size of the AuNPs was measured by transmission electron microscopy (TEM) analysis, and they were stored at 4°C in a glass vial for future use.

### Synthesis of Gold Nanoparticle Assemblies

In a typical process, 0.1 ml of AuNP solution was dispersed in 0.9 ml of DI water, followed by the addition of an aqueous solution of APTES (5 μl, V_APTES_:V_EtOH_ = 1:10). The assembly process could be clearly observed by the color change in the solution from wine red to bluish purple.

### Disassembly of Gold Nanoparticle Assemblies

Typically, 10 μl of NaOH solution (0.5 M) was added into the dispersion of AuNP assemblies under manual shaking. A UV-vis spectrometer (HR2000+CG-UV-NIR, Ocean Optics) was used to measure the real-time spectra changing during the disassembled process.

### Assembly of Gold Nanoparticles

HCl solution (10 μl) (0.5 M) was added into the disassembled dispersion of AuNP assemblies under manual shaking. The UV-vis spectrometer (HR2000+CG-UV-NIR, Ocean Optics) was also used to record the changing of the real-time spectra during the re-assembled process.

### Characterization

The optical properties of AuNPs and AuNP assemblies were measured by a UV-Vis spectrophotometer (HR2000+CG-UV-NIR, Ocean Optics). The morphology of AuNP assemblies was performed on a transmission electron microscope (TEM, Hitachi HT7700) operated at 100 kV. Fourier transform infrared (FTIR) spectra were recorded on a Bruker ALPHA II spectrophotometer scanning from 4,000 to 400 cm^−1^ with a resolution of 4 cm^−1^. Dynamic light scattering (DLS) measurements were recorded on a zeta-potential and particle size analyzer (Z3000, Nicomp Particle Sizing Systems).

## Results and Discussion

**Figure 1:**
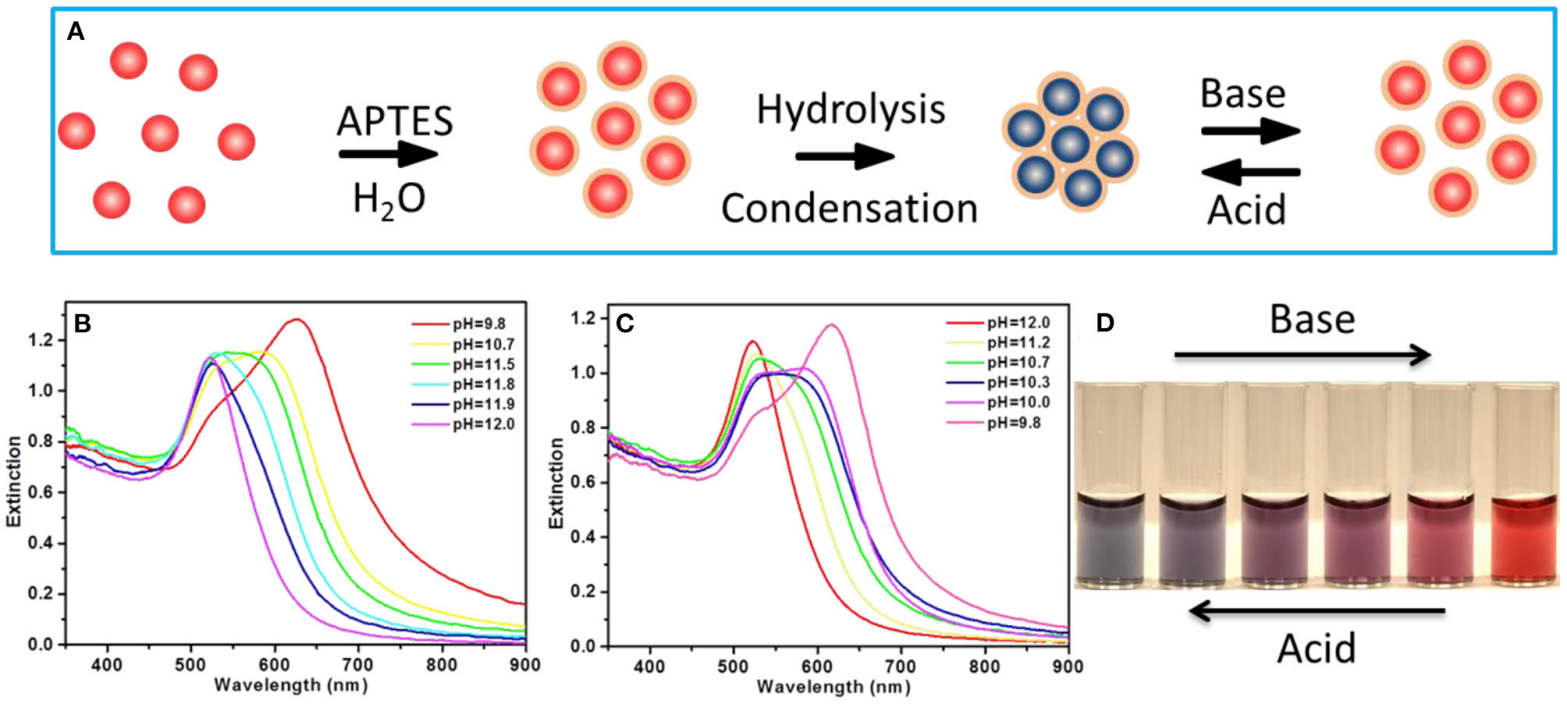
**(A)** Schematic illustration of the reversible assembly of Au nanoparticles (AuNPs). **(B)** The UV-vis extinction spectra of the AuNP assemblies with pH increasing from 9.8 to 12.0. **(C)** The UV-vis extinction spectra of the AuNP assemblies with pH decreasing from 12.0 to 9.8. **(D)** The digital photo of the AuNP solution showing color evolution with pH variation.

The citrate-capped AuNPs with a diameter of 15 nm were synthesized by using the classic citrate reduction method and used in this paper for reversible assembly (Figure 1A). Once APTES was added into the Au solution, the amino group of APTES bonded with the AuNPs; dispersive AuNPs then proceeded into self-assembly owing to the hydrolysis of APTES, where the color of the solution changed from wine red to blue accompanied by a shoulder peak appearing at a longer wavelength around 650 nm (Supplementary Figure 1). APTES on the AuNP surface can be dynamically manipulated by pH variation, thereby enabling reversible assembly of AuNPs. The optical behavior of reversible assembly was also monitored as a function of pH using a UV-vis spectrometer. It was clear to see that upon increasing pH from 9.8 to 12.0, the longer wavelength peak was blue shifted and eventually went back to the intrinsic peak of AuNPs at 520 nm (Figure 1B), with the color change in solution from blue, bluish purple, purple to wine red (Figure 1D), while the optical properties of AuNP assemblies present an inverse phenomenon with the pH decreasing from 12.0 to 9.8 (Figure 1C), accompanied by a solution color change from wine red, purple, bluish purple to blue (Figure 1D).

**Figure 2:**
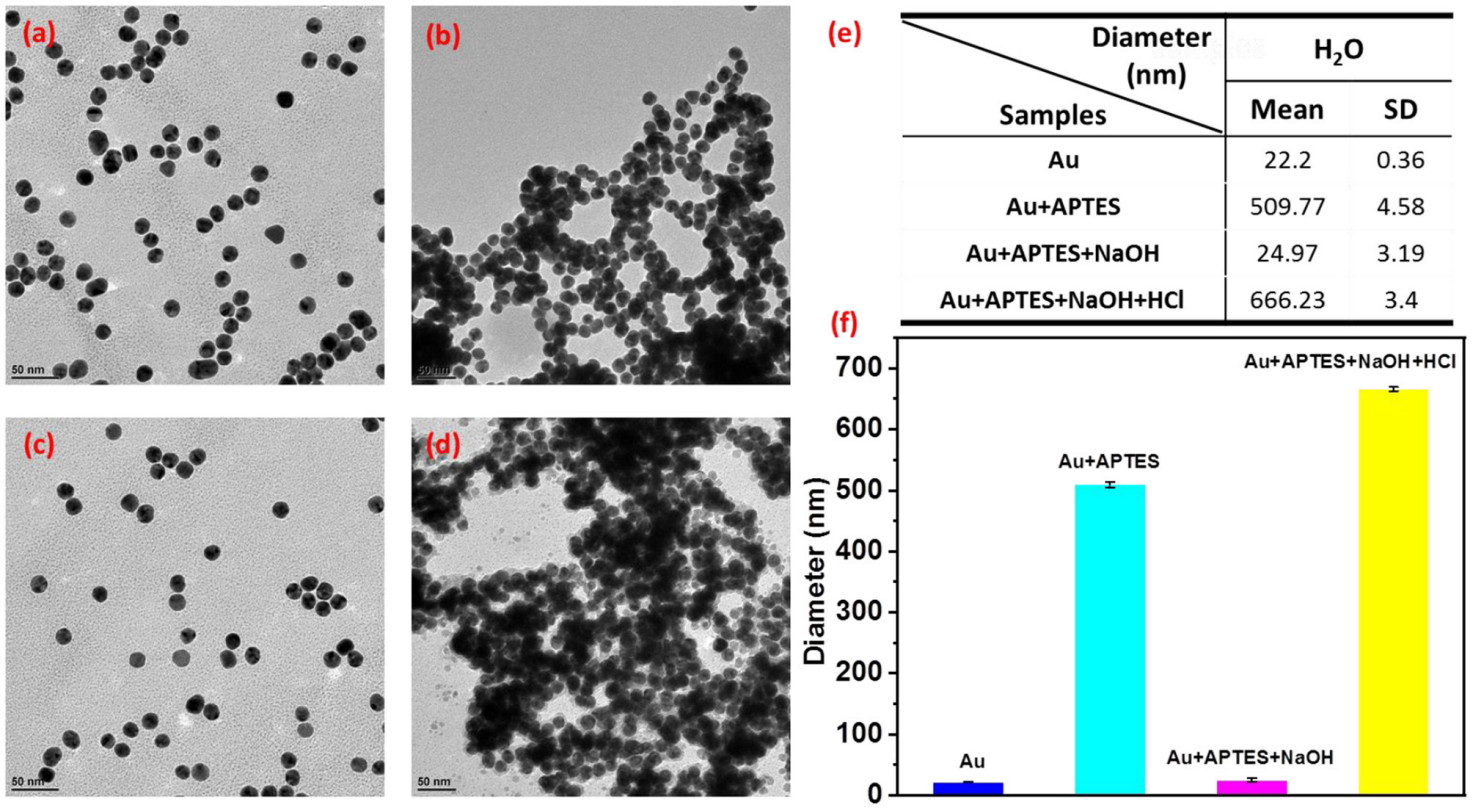
Transmission electron microscopy (TEM) images of monodisperse AuNPs **(a)**, the dispersion of AuNPs by adding 3-aminopropyltriethoxysilane (APTES) **(b)**. The disassembly of AuNP assemblies with pH increasing from 9.8 to 12.0 **(c)**, and the re-assembly of AuNPs with pH decreasing from 12.0 to 9.8 **(d)**. **(e,f)** The size of AuNP assemblies and discrete AuNPs by dynamic light scattering (DLS).

The morphologies of the reversible assembly of AuNPs were characterized by transmission electron microscope (TEM). Figure 2a shows that the isolated spherical AuNPs with an average size of ~15 nm were successfully formed by using the classic citrate reduction method; then, the AuNPs gradually assembled into aggregates with increasing sizes after the addition of APTES (Figure 2b), which agreed with the UV-vis spectra data (Supplementary Figure 1). In the reversible assembly process, the AuNP assemblies would be disassembled into smaller aggregates, eventually forming discrete nanoparticles with an increase in the solution pH to 12.0 (Figure 2c), while the nanoparticles could be re-assembled with a size increase by decreasing the pH to 9.8 (Figure 2d). The disassembly and re-assembly behavior was further inspected by the dynamic light scattering (DLS) measurements conducted by adding different volumes of NaOH and HCl solutions into the solution of AuNP assemblies (Figures 2e,f). The average size of the AuNP assemblies decreased from about 509.77 ± 4.58 to 24.97 ± 3.19 nm during disassembly, while the average size increased from 24.97 ± 3.19 to 666.23 ± 3.4 nm during re-assembly, which agreed well with the TEM.

**Figure 3:**
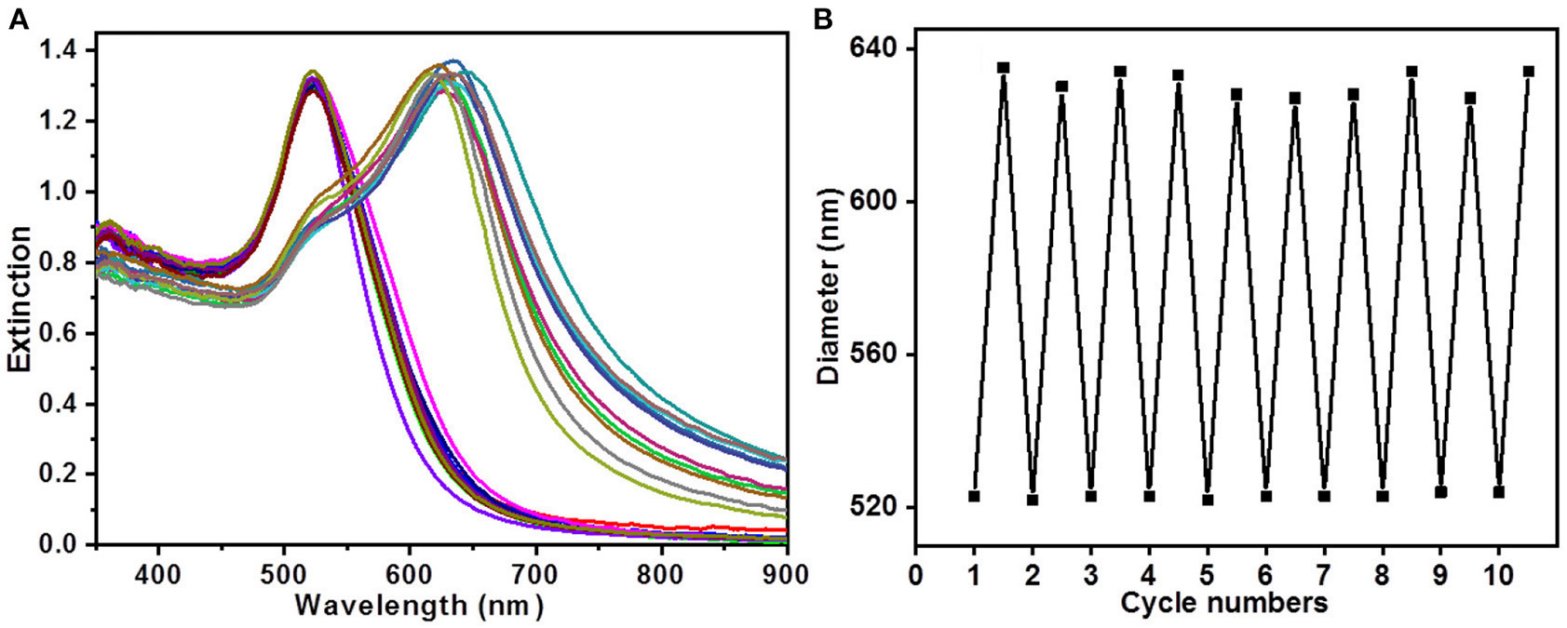
**(A)** UV-vis spectra of 10 cycles switching between disassembled and assembled states. **(B)** Repeated changes of plasmonic peak positions in the 10 switching cycles.

To investigate the stability and reversibility of the AuNP assemblies, the system was cycled 10 times by alternately adding NaOH and HCl into solution. As shown in the UV-vis spectra in Figure 3A, two absorption spectra corresponding to the extinction profiles could be switched by adding NaOH and HCl into the dispersion. Figure 3B plotted the peak positions of the coupled surface plasmon of the dispersion with the addition of NaOH and HCl, respectively, which demonstrated impressive reversibility and reproducibility of the AuNP assemblies.

**Figure 4:**
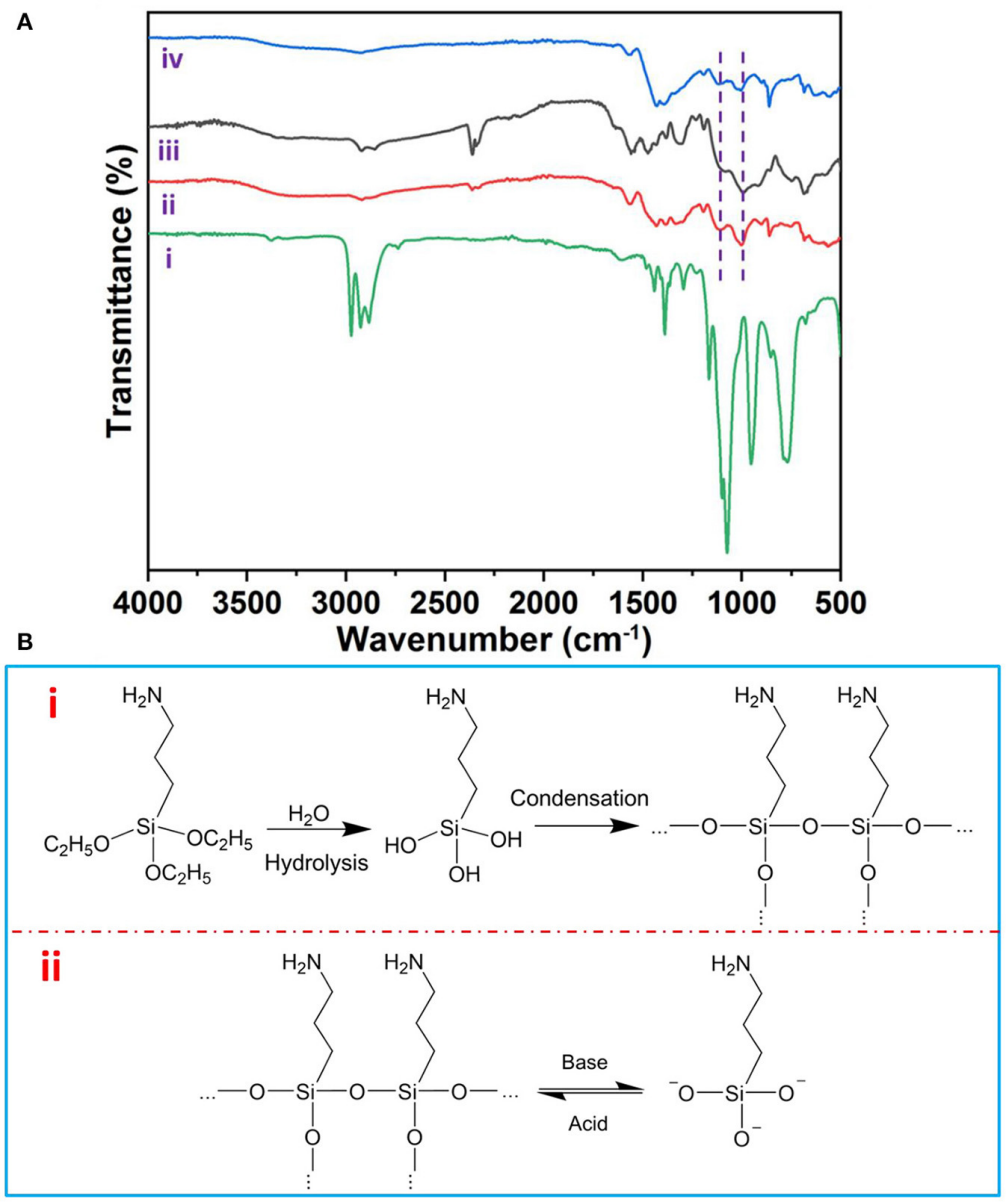
**(A)** Fourier transform infrared (FTIR) spectra of APTES (i), initial (ii), disassembled (iii), and re-assembled AuNP assemblies (iv). **(B)** The mechanism of pH-responsive reversible assembly of AuNPs.

As shown in Figure 4A, Fourier transform infrared spectra (FTIR) detailedly revealed the asymmetric stretching modes of Si–O–Si bond peaking at 1,130 and 1,044 cm^−1^ (Majoul et al., 2015) on the surface of AuNPs, which could reversibly disappear and reappear upon the addition of NaOH and HCl solution, respectively. Therefore, we can draw the reversible assembly mechanism as follows: (i) APTES acts as the capping ligand bonded with AuNPs *via* the amino group of APTES (Feng et al., 2019). (ii) the APTES on the AuNPs' surface plays a key role as a pH-responsive agent; the APTES itself hydrolyzes rapidly in response to acid and then condenses into silicon (De et al., 2000). On the contrary, the condensed silicon can be broken down into silicate by base (Zhang et al., 2008), which subsequently deprotonate the APTES on AuNPs (Figure 4B).

## Conclusions

In summary, we have developed a novel method for direct reversible assembly of AuNPs in water driven by pH stimuli with robust dynamic tuning of the surface plasmonic coupling among AuNPs. APTES plays a critical role in the system, as the capping ligand bonded with AuNPs *via* the amino group of APTES. Moreover, the APTES on the AuNPs' surface endows highly reversible assembly and dynamic color change to the system as the Si–O–Si bonds can be reversibly manipulated by controlling pH variation. Compared with the previous Au-based system, our system provides a facile and distinctive strategy exhibiting significant advantages such as higher color contrast, simpler procedure, better reversibility and reproducibility, and lower cost. In addition, this study also offers a new avenue for other plasmonic nanoparticles to adjust plasmonic properties *via* reversible assembly.

## Data Availability Statement

The original contributions presented in the study are included in the article/Supplementary Material, further inquiries can be directed to the corresponding author.

## Author Contributions

YL and WF designed and conducted some of the experiments, and wrote this paper. ZX, LZ, TS, and MD did the main experiment works. XK and SX processed some of the data. CZ processed the figures. MG and DZ reviewed and improved the paper.

## Conflict of Interest

The authors declare that the research was conducted in the absence of any commercial or financial relationships that could be construed as a potential conflict of interest.
