# Supplementary material for: pH-Driven Reversible Assembly and Disassembly of Colloidal Gold Nanoparticles
Source: Front Chem. 2021 Apr 29;9:675491. doi: 10.3389/fchem.2021.675491 (PMC8116534; doi:10.3389/fchem.2021.675491)
Supplement: Supplementary file 1 [file Image_1.pdf]

pH-Driven Reversible Assembly and Disassembly of Colloidal Gold Nanoparticles

Yun Liu[1]*, Weihua Fu[2], Zhongsheng Xu[1], Liang Zhang[1], Tao Sun[1], Mengmeng Du[1], Xun Kang[1], Shilin Xiao[1], Chunyu Zhou[1], Mingfu Gong[1]*, Dong Zhang[1]*

[1]Department of Radiology, Xinqiao Hospital, Army Medical University, Chongqing 400037, China

[2]Department of Urology, Xinqiao Hospital, Army Medical University, Chongqing 400037, China

*Corresponding Author: Yun Liu

E-mail address: yunliu@tmmu.edu.cn (Yun Liu)

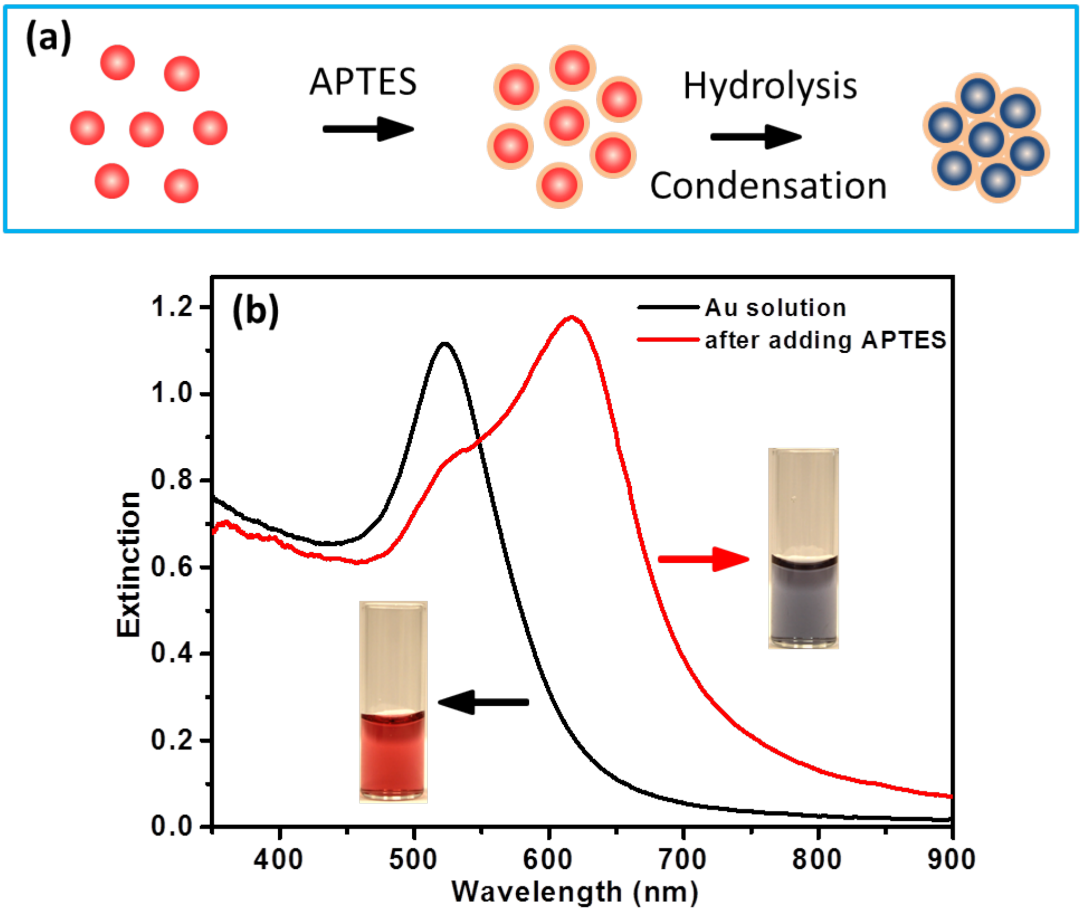

Figure S1. (a) Schematic illustration of the assembly of AuNPs with the addition of APTES. (b) The UV-vis extinction spectra of the initial AuNPs solution with and without adding APTES (The inset is the digital photos of the AuNPs solution with and without adding APTES).
